# Dialysate temperature adjustment as an effective treatment for baroreflex failure syndrome in hemodialysis patient

**DOI:** 10.1186/1471-2369-15-151

**Published:** 2014-09-17

**Authors:** Natsumi Tanabe, Koki Takane, Keitaro Yokoyama, Yudo Tanno, Izumi Yamamoto, Ichiro Ohkido, Takashi Yokoo

**Affiliations:** Jikei University Hospital, 3-25-8, Nishishimbashi, Minato-ku, Tokyo, 105-0003 Japan; The Division of Nephrology and Hypertension, Jikei University School of Medicine, 3-25-8, Nishishimbashi, Minato-ku, Tokyo, 105-0003 Japan

**Keywords:** Baroreflex failure, Hemodialysis, Hypertension

## Abstract

**Background:**

Baroreflex failure syndrome is a rare disorder which causes labile blood pressure, headache, flushing, diaphoresis and emotional lability. It is caused by history of trauma or radiotherapy in the cervical legion, bilateral carotid-body tumor or resection of glossopharyngeal nerve. We experienced a case of hemodialysis patient who had difficulty in controlling blood pressure during dialysis because of his baroreflex failure syndrome and successfully controlled his blood pressure by adjusting dialysate temperature.

**Case presentation:**

We report a case of a 68-year-old CKD5 patient who had difficulty in hemodialysis treatment because of severe fluctuations in blood pressure with hypertensive attacks and hypotensive episodes which caused him a severe discomfort. His dialysis treatment was started in 2010 and since that time baroreflex failure syndrome has been suspected because of his clinical manifestations and history of radiotherapy in the cervical region for his lingual cancer in 1994. Baroreflex failure syndrome is diagnosed by symptoms and cold stressor test. We performed a cold stressor test on an experimental baroreflex failure syndrome mouse and induced a significant elevation of blood pressure. From this experimental finding of model mouse, we changed the patients dialysate temperature between 34-38° according to his change in blood pressure though 80–240 mmHg. From this attempt, his blood pressure was successfully controlled between 100–180 mmHg and he was able to continue hemodialysis without any discomfort.

**Conclusion:**

In our case, environmental stimulation such as temperature change modified the patients fluctuating blood pressure. Change of dialysate temperature could be an option for controlling the unstable blood pressure due to baroreflex failure syndrome.

## Background

The arterial baroreflex which involves multiple components of the baroreflex arc, prevents excessive fluctuations of arterial blood pressure.

The signals by distention of the vessel wall are sent from baroreceptors in each carotid sinus to the brain stem via the glossopharyngeal nerve (cranial nerve IX). The information from other baroreceptors in the aortic arch and the great vessels of the thorax are sent through the vagus nerve (cranial nerve X) to the brain stem. Thoracic blood volume change detected by low-pressure receptors is also sent by the vagus nerve to the brain stem.

According to these mentioned mechanism of baroreflex, any abnormalities in the vascular baroreceptors, the glossopharyngeal or vagus nerves, or the brain stem could cause baroreflex failure [1].

**Figure 1:**
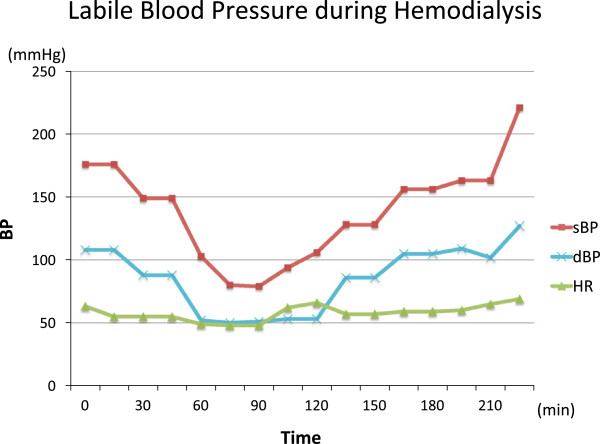
**Fluctuating blood pressure during hemodialysis without changing the dialysate temperature.** This is the hemodynamic change during convectional dialysis before applying the temperature adjusting dialysis to the patient. The systolic blood pressure during hemodialysis changed through 80 mmHg to 240 mmHg before we started the dialysate temperature therapy. The patient suffered from discomfort and diaphoresis from this blood pressure change.

In clinical setting, baroreflex failure is often caused by denervation of carotid body tumor resection, carotid artery surgery, neck irradiation and neck trauma. The frequent symptoms are labile hypertension and hypotension, often with headache, diaphoresis, face flashing and emotional instability.We experienced a hemodialysis case with labile blood pressure which fluctuated through 80 mmHg to 240 mmHg during dialysis (Figure 1).

Although it was hard to diagnose, we differentiated our case from autonomic failure, which is common in chronic hemodialysis patients, by the hyperactive result to cold pressure stimuli. This is because sympathetic efferents to the vasculature are intact in baroreflex failure and patients exhibit a normal or even an increased pressor response to cold-pressure test [2].

In general, the use of low-temperature dialysate is recommended to decrease the frequency and intensity of symptomatic hypotension. From this evidence, we considered applying this method to stabilize the patients’ labile blood pressure.

This is a case report of baroreflex failure hemodialysis patient in whom dialysate temperature adjustment was an effective treatment for labile blood pressure during hemodialysis caused by baroreflex failure syndrome.

## Case presentation

### Case

**Figure 2:**
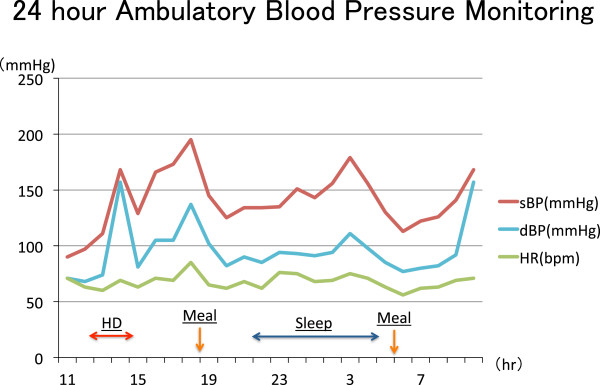
**The data of 24 hour ambulatory blood pressure monitoring (24 hr ABPM).** This is the data of 24 hour ABPM on dialysis day. There was an extreme change in blood pressure during the day and the patient experienced hypotensive episodes several times. Most episodes were after inserting meal from PEG and after lying in bed, when vagus nerve was stimulated. These episodes also indicate that the patient was unable to have vasovagal reflex when there was vagus nerve stimulation.

A 68-year-old Danish male presented with multiple episodes of lightheadedness and the feeling of “passing out” while he was getting into his bed or when food was administered to his gastric fistula tube. And he also had sudden onset of hypertensive attacks several times a day. His past medical history was significant for chronic kidney disease and hypertension since he was 30-years-old. The patient was diagnosed having lingual cancer for which he received external radiation therapy (RT) at 51-year-old. Ten years after RT, the patient started having dysphagia and had several episodes of aspiration pneumonia. Modified barium swallow showed he was having silent aspiration and Percutaneous Endoscopic Gastrostomy (PEG) was created. Although the patient didn’t have any oral intake, he still repeated pneumonia and eventually he underwent tracheotomy. At the same time, his renal function declined and hemodialysis was started three times a week. Since then he has been suffering labile blood pressure.During dialysis, interdialytic weight increase was controlled between 1.5–1.7 kg (2-3% of body weight) and ultrafiltration rate was about 6.0 – 6.3 ml/hr/kg. The patients’ sodium concentration was mostly controlled between 140 to 142 mEq/L and diffusive sodium balance during dialysis was negative. Although this stable weight and sodium control, blood pressure fluctuated through 80 mmHg to 240 mmHg especially during dialysis and was difficult to control with medications (Figure 1).His daily blood pressure had been treated by Ca-channel blocker and ARB but the labile blood pressure could not be stabilized and he had frequent episodes of fainting and several occlusion of his arteriovenous fistula (AVF) caused by extreme hypotension. Everyday, there were several timings when the blood pressure dropped. Most of the time it was after inserting food from the PEG and sometimes after lying in bed (Figure 2). His current medication included Droxidopa and Midodrine Hydrochloride for hypotensive episodes but his labile blood pressure was difficult to control with these medications especially during hemodialyisis.

The dialysate had the following ionic concentration: 140 mEq/L of sodium, 2.0 mEq/L of potassium, 3.0 mEq/L of calcium, 1.0 mEq/L of magnesium, 113 mEq/L of chloride, and 10 mEq/L of acetate.

Although his blood pressure was extremely unstable, heart rate was stable and didn’t change more than 10 bpm during hemodialysis.

Eventually, fifteen years after the neck irradiation, this patient suffered from edema of his arm with his AVF. It was caused by the stenosis of his brachial vein and cured by Percutaneous Transluminal Angioplasty (PTA).

**Figure 3:**
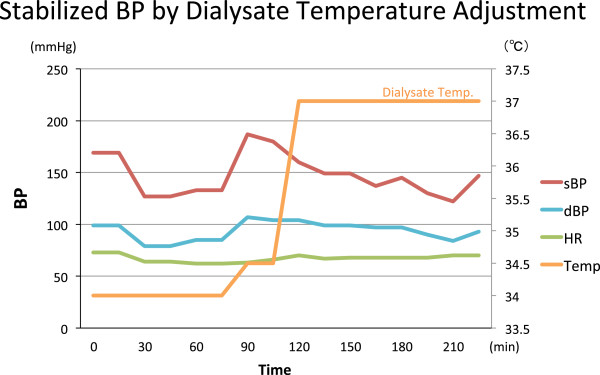
**Less fluctuating blood pressure during hemodialysis with dialysate temperature therapy.** After starting the dialysate temperature adjustment therapy, blood pressure was less fluctuating and remained between 120 mmHg to 180 mmHg. This figure shows the association between the dialysate temperature change and the blood pressure change.

From his current symptom of labile blood pressure with stable heart rate and medical history of RT and brachial vein stenosis, we diagnosed him having Baroreflex Failure.The patient’s labile blood pressure during hemodialysis was treated with dialysate temperature change. We decreased dialysate temperature to 34° when patient’s blood pressure was low and increased it to 37° when pressure was getting high. From this trial, his blood pressure was stabilized between 120–180 mmHg which was ranging through 80–240 mmHg before the temperature adjustment (Figure 3). Similar to convectional dialysis without temperature adjustment, the diffusive sodium balance during temperature adjusting dialysis was negative and the ultrafiltration rate was 6.16 ml/hr/kg.

### Examination

His physical exam was negative except his hypertension. Extensive laboratory examinations did not show any abnormal findings. Cold pressor test showed 26 mmHg, 15 mmHg increase in sBP, dBP, respectively [2]. During the day, there were several timings of blood pressure drop, which was after meals and after lying in bed (Figure 2).

### Pathophysiology

At the beginning of every hemodialysis, the patients’ BP was always higher than normal range with his systolic blood pressure around 170 mmHg and it gradually dropped to around 80 mmHg in the first one hour of hemodialysis. After reaching the bottom it kept on rising to 240 mmHg toward the end of the dialysis.

Several factors causing intra-dialysis hemodynamic instability have been reported in many articles in the past. Inter-dialytic water and sodium control predominantly affects the intra-dialysis hemodynamic instability [3]. Other than that, sodium concentration change, ultrafiltration rate, activation of RAA or sympathetic activity during hemodialysis are the causes of intra-dialysis hemodynamic instability [4].

In this case, inter-dialytic weight increase was controlled between 1.5–1.7 kg (2-3% of body weight). The dialysate sodium level was 140 mEq/L and the patients’ pre dialysis plasma sodium level was mostly maintained between 140 mEq/L to 142 mEq/L. (The dialysis was done three times a week in the afternoon. The sodium was measured pre and post dialysis from the site of paracentesis. In order to remove the dialysate affection, sufficient amount of blood was drawn before the sample taken). According to the lab results during dialysis, the diffusive sodium balance during dialysis was negative and the ultrafiltration rate was 6.30 ml/hr/kg. The transition of lab results during convectional dialysis without temperature adjustment is shown below (Table 1).

**Table 1:** **The various parameter change during convectional hemodialysis without temperature adjustment**

**Time from starting HD (min)**	0	60	120	180
**sBP (mmHg)**	150	109	125	210
**dBP (mmHg)**	97	74	88	110
**HR (bpm)**	66	61	65	77
**Body Weight change (kg)**	73.9			72.3
**PRA (ng/ml/hr)**	0.3	0.3	0.2	0.3
**PAC (pg/ml)**	91.6	45.6	52.9	48.4
**Adrenaline (pg/ml)**	20	32	20	40
**Noradrenaline (pg/ml)**	158	147	208	227
**Dopamine (pg/ml)**	38	38	28	26
**Hct (%)**	34.3			35.8
**Na (mEq/L)**	140			139
**K (mEq/L)**	4.1			3.5
**Ca (mEq/L)**	9.2			10.0
**Plasma Osml (mOsm)**	305			287
**hANP**	111			73.8

From these results, the patients hemodynamic instability during dialysis was not thought to be caused by volume overload nor sodium imbalance.

In the process of making the diagnosis, we noticed that the patients’ heart rate did not change and was stable compared to his blood pressure instability. From his past medical history of RT and unusual blood pressure elevation in cold pressor test, we diagnosed him having baroreflex failure.

**Figure 4:**
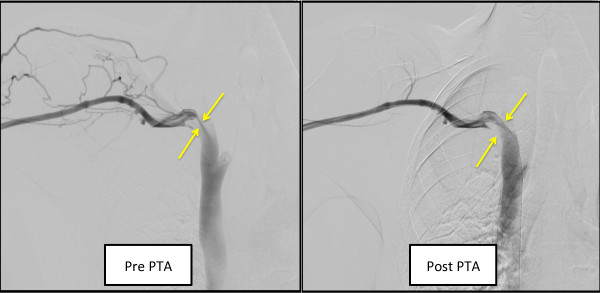
**A fluoroscopic image of the patients occluded brachial vein, pre and post PTA.** The patient experienced an occlusion of the brachial vein 15 years after the neck irradiation. We presume that there was a fibrotic change in the brachial vein from the neck irradiation. We performed PTA and the fibrotic brachial vein was successfully dilated.

The key for diagnosing this case was his medical history of RT in the cervical region. Among several causes of baroreflex failure, a history of irradiation around the cervical region is one of the main reasons [5]. Since the average duration of time until symptomatic is 5–15 years, it is often seen as a chronic complication in RT. In particular, after neck irradiation, long-term injury occurs commonly in the carotid arteries. Atherosclerotic and thrombotic complications occur and cause significant carotid stenosis and stroke in many patients. In fact, this patient had medical history of TIA ten years after the irradiation. Besides atherosclerosis, chronic inflammation and fibrosis of carotid arterial walls might lead to “splinting” of carotid sinus baroreceptors. Since these receptor are sensitive to stretch or distortion, stiffening of the carotid sinus would be expected to lead to decreased gain of the arterial baroreflex [6]. In this case, Right Internal Carotid Artery (R-ICA) starting point and Left common carotid artery (L-CCA) bifurcation points Max Intima-media Thickness (IMT) was 3.41 mm homogenous iso-echoic plaque and 3.68 mm heterogenous iso-echoic plaque, respectively. While hypo echoic plaque is usually compatible to hematoma or atheroma, and hyper echoic plaque is usually reflect calcification, homogenous iso-echoic plaque is often seen in case of the fibrosis of the artery [7].In addition, three years after starting the temperature adjustment dialysis, this patient suffered from edema of his arm which he had his AVF. It was caused by the stenosis of his brachial vein and cured by Percutaneous Transluminal Angioplasty (PTA) (Figure 4).

By the facts that Carotid Ultrasonography showed iso-echoic plaques and he had a brachial vein stenosis which was cured by PTA, it was compatible to our thought that the fibrosis of the artery is main reason to cause his baroreflex failure after radiation therapy.

The recommended medication for baroreflex failure is antihypertensive and/or vasopressor agents to prevent labile change in blood pressure [5]. Our patient took droxidopa, which was somewhat effective, before meal and lying in bed because these two situations caused hypotensive episodes. However the labile blood pressure during hemodialysis was not able to be stabilized by medications.

**Figure 5:**
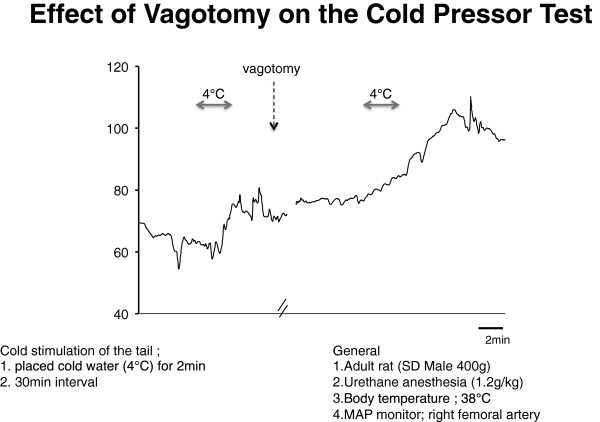
**Cold pressor test on vagotomy model mouse.** This figure shows the systolic blood pressure change of the vagotomy model mouse during cold pressor test. Vagotomy mouse, which is a model of baroreflex failure, has more increase in blood pressure than normal mouse when the tail was exposed in 4° water for 2 minutes. This experiment shows that the blood pressure of baroreflex failure model mouse is more susceptible to cold stimulation.

The 2005 K/DOQI guidelines [8] recommend the use of cool dialysate temperature dialysis in patients with frequent episodes of uncontrolled hypotension during hemodialysis treatment. Although blood pressure during dialysis is mostly affected by diffuse sodium balance and ultrafiltration rate, peripheral temperature stimulation could change the blood pressure from other pathway. Since the peripheral nerve is intact in baroreflex failure, we decided to apply this method to our case. And to reveal the temperature susceptibility in baroreflex failure, we performed a cold pressor experiment on vagotomy model mouse. From the experiment, vagotomy mouse, which is a model of baroreflex failure, have more increase in blood pressure than normal mouse when the tail was exposed into 4° water for 2 minutes (Figure 5). This suggests the high susceptibility to temperature change in baroreflex failure. And in addition to this, our patient used more dose of droxidopa during summer than during winter. From these findings, we considered the blood pressure of baroreflex failure responds more to temperature change than usual hemodialysis patients.

In our case the diffuse sodium balance was negative and ultrafiltration rate was 6.30 ml/hr/kg and 6.16 ml/hr/kg in convectional dialysis (Figure 1) and temperature adjusting dialysis (Figure 3), respectively. This diffuse sodium balance and ultrafiltration rate was very similar between the two. Negative diffuse sodium balance may affect the RAAS pathway and lead to blood pressure change, but during both dialysis, the Plasma Renin Activity (PRA) did not change extremely and the Plasma Aldosterone Concentration (PAC) declined toward the end of the dialysis (Tables 1 and 2). From these results, the previous hemodynamic instability during dialysis was not likely to be caused by volume change nor RAAS effect but possibly cased by the overstimulation of autonomic nerve with no vagal reflex.

**Table 2:** **The various parameter change during hemodialysis with temperature adjustment**

**Time from starting HD (min)**	0	60	120	180
**sBP (mmHg)**	178	132	157	165
**dBP (mmHg)**	103	77	91	112
**HR (bpm)**	67	58	59	71
**Body Weight change (kg)**	74.6			72.9
**PRA (ng/ml/hr)**	0.6			0.5
**PAC (pg/ml)**	94.2			44.9
**Adrenaline (pg/ml)**	17			40
**Noradrenaline (pg/ml)**	297			144
**Dopamine (pg/ml)**	88			25
**Na (mEq/L)**	141			140

From this negative diffuse sodium balance and ultrafiltration rate similarity between convectional and temperature adjusting dialysis, we presume that the blood pressure was successfully controlled through peripheral temperature stimulation in temperature adjusting dialysis.

From our case, we considered baroreflex failure patient have more susceptibility to temperature change than usual hemodialysis patient and temperature adjusting dialysis is an effective way to control blood pressure in these patients.

## Conclusion

Baroreflex failure occurs as a chronic complication for neck irradiation therapy. Patients who have baroreflex failure often suffer from their unstable blood pressure. Baroreflex failure dialysis patient who had an extreme change in blood pressure during dialysis was treated successfully by adjusting the dialysate temperature according to the labile blood pressure during hemodialysis.

### Consent

Written informed consent was obtained from the patient for publication of this Case report and any accompanying images. A copy of the written consent is available for review by the Editor of this journal.

## Abbreviations

CKD: Chronic kidney disease
RT: Radiation therapy
PEG: Percutaneous endoscopic gastrostomy
AVF: Arteriovenous fistula
PTA: Percutaneous transluminal angioplasty
sBP: Systolic blood pressure
dBP: Diastolic blood pressure
RAA: Renin angiotensin aldosteron.
